# Conferences/Events: WORKSHOP ON MICROSTRUCTURE AND MACROMOLECULAR RESEARCH WITH COLD NEUTRONS National Bureau of Standards, Gaithersburg, MD, April 21–22, 1988

**DOI:** 10.6028/jres.093.161

**Published:** 1988-12-01

**Authors:** C. J. Glinka, J. A. Gotaas, C. O’Connor

**Affiliations:** Institute for Materials Science and Engineering National Institute of Standards and Technology Gaithersburg, MD 20899

On April 21–22, 1988, over 160 scientists, including 25 from industry, 70 from universities and 30 from government laboratories and agencies (not including NBS), gathered at NBS, Gaithersburg, for a Workshop on Microstructure and Macromolecular Research with Cold Neutrons. This workshop was one of a series, each devoted to a major area of research with cold neutrons, that are being held in conjunction with the development of the Cold Neutron Research Facility (CNRF) at the NBS 20 MW research reactor as a national, user-oriented research facility. The dual aims of this workshop were to highlight research opportunities in the application of cold neutron techniques to the study of submicron structure in materials and macromolecular systems, and to inform and involve the scientific community in the planning for instrumentation for the CNRF.

The long wavelength neutrons that are produced by a cold moderator, such as the 40 K D_2_O-ice moderator recently installed in the NBS reactor, are a powerful probe of both the structure and dynamics of materials. The emphasis of this workshop was on the structural applications of cold neutrons. Thus techniques such as small angle neutron scattering (SANS), which probes structure in bulk materials on the 1 to 100 nm scale, and the relatively new technique of neutron reflectometry, which probes near surface structure to depths of tenths of microns, were discussed in detail. For both of these techniques, cold neutrons substantially extend the distance scale probed and improve the spatial resolution beyond what has been possible with thermal neutrons.

The first morning session of the workshop on Scientific Opportunities with Cold Neutrons opened with a review by T. Russell (IBM Almaden Research Center) of the impact SANS has had on Polymer Science in general, and on the understanding of the interactions and correlations in polymer mixtures in particular. Russell also discussed recent developments, important to the development of polymer composites, in the use of grazing incidence neutron and x-ray scattering for the evaluation of conformation changes and concentration gradients in polymer mixtures near surfaces and at interfaces. The use of scattering methods to characterize such seemingly dissimilar materials as porous rock and agglomerated colloids was the subject of the talk by S. K. Sinha (Exxon Research and Engineering Co.). Sinha presented a general formalism which unified the concepts of surface and mass fractals and showed how the self-similar nature of fractal structures is most directly revealed through scattering measurements. Several examples were presented which underscored the need for scattering instruments which probe a wide range of length scales in order to accurately determine the nature and limits of fractal behavior.

The second half of this session was devoted to applications of neutron reflectometry, reviewed by R. K. Thomas (Oxford University), and grazing incidence diffraction, covered by H. Zabel (University of Illinois). Thomas discussed the complementarity of neutron and x-ray reflection techniques for studying inhomogeneities normal to a surface or interface. He gave several examples of reflection measurements on surfactants and polymers adsorbed at the surfaces of solutions which demonstrated the use of contrast variation to enhance the scattering from the adsorbed layer, which is the particular advantage of the neutron technique. In his talk, H. Zabel outlined the basic theory of the novel technique of surface dynamical diffraction and reflection and presented the first neutron results obtained for a Si(110) surface. This technique, which exploits the interplay between simultaneous Bragg diffraction and surface reflection to provide structural information both parallel and normal to a surface, is potentially a powerful probe of surface reconstruction, roughening transitions, and the nature of surface magnetic structures.

The first afternoon session was devoted entirely to reviewing the current status and future plans for the NBS CNRF. NBS Deputy Director Ray Kammer opened the session by outlining the overall scope of the CNRF as a national user facility open to all qualified researchers on the basis of scientific merit. He went on to describe the user policy for the facility and the plan for instrument development which allows for one-third of up to 15 experimental stations to be developed by groups outside NBS, so-called Participating Research Teams, who would then receive three-fourths of the available beam time. Kammer’s policy overview was followed by a technical overview by J. M. Rowe of NBS who described the architectural design of the experimental hall and office wing (scheduled for completion in early 1989) associated with the CNRF, and the network of totally reflecting guide tubes that will transport neutrons from the reactor cold source to the CNRF. He also presented data on the performance of the D_2_O-ice cold source now in operation in the reactor and gave a timetable for projected instrument development.

The remaining four talks in this session each focused on a specific cold neutron technique and presented design concepts for its implementation in the CNRF. Described were a novel SANS instrument that would utilize a doubly curved mirror to focus a beam onto a detector, the current state of development of neutron supermirrors and their use both to enhance flux and to produce polarized neutron beams, a neutron reflectometer suitable for measurements on both solid and liquid surfaces, and an improved facility for neutron depth profiling which would utilize a converging neutron guide to increase the sensitivity of this technique by more than one order of magnitude over what is now possible using thermal neutrons.

The application of cold neutron methods to the study of submicron structure in specific classes of materials was the theme of the second morning session of the workshop. J. Hayter (Oak Ridge National Laboratory) opened the session with a survey of submicron chemical systems such as colloids and micellar solutions whose structure and interactions can be probed effectively with cold neutrons, while G. Zaccai (Institut Laue-Langevin) gave a corresponding survey of applications to biological systems such as protein solutions and DNA-complexes. Metallurgical applications, with an emphasis on time-resolved studies of phase separation in binary alloys, were reviewed by B. Gaulin (Oak Ridge National Laboratory). Finally, R. Page (Southwest Research Institute) gave a critical review of current and potential applications in the study of damage accumulation, phase transformations, and processing of advanced ceramics.

The final afternoon of the workshop consisted of three discussion sessions which provided participants with the opportunity to comment upon or inquire further about any aspect of the CNRF. The first session on Macromolecular Dynamics explored the potential impact which cold neutron inelastic scattering techniques could have in the study of molecular motions in macromolecular systems. C. Han of the NBS Polymers Division along with Z. Akcasu and S. Krimm, both of the University of Michigan, stimulated the discussion by giving their views of the important scientific issues which could be addressed with advanced neutron scattering instrumentation. This discussion flowed naturally into the next session on Instrumentation and Techniques in which progress in several key areas of cold neutron instrumentation were highlighted. The final session on User Needs focused on the practical needs of the potential user of the CNRF. This discussion was wide-ranging and touched on security requirements for access to the facility, proposal procedures, travel support, housing availability, etc. Several participants stressed the need for adequate sample preparation facilities particularly for biological samples which often have limited lifetimes and thus cannot be prepared far in advance.

One clear impression that emerged from the workshop is that there is a strong and growing interest in the scientific potential of the relatively new techniques of neutron reflectometry and grazing incidence diffraction for the study of near surface and interface structure. As a result of this expressed interest, the development of a state-of-the-art neutron reflectometer for the CNRF will be given increased priority. This instrument, along with a 30 m SANS machine, are expected to be the first instruments to go into operation when the CNRF guide hall is completed.

Copies of the workshop program containing the abstracts of the invited talks, and a 12-page booklet describing the CNRF may be obtained by contacting Carol O’Connor, National Institute of Standards and Technology, Bldg. 235, Rm. A106, Gaithersburg, MD 20899, 301/975-6240.

